# Metabolomics-Driven Exploration of the Antibacterial Activity and Mechanism of 2-Methoxycinnamaldehyde

**DOI:** 10.3389/fmicb.2022.864246

**Published:** 2022-07-07

**Authors:** Chunguo Qian, Lu Jin, Longping Zhu, Yang Zhou, Jing Chen, Depo Yang, Xinjun Xu, Ping Ding, Runnan Li, Zhimin Zhao

**Affiliations:** ^1^School of Pharmaceutical Sciences, Sun Yat-sen University, Guangzhou, China; ^2^Guangdong Technology Research Center for Advanced Chinese Medicine, Guangzhou, China; ^3^School of Pharmaceutical Science, Guangzhou University of Chinese Medicine, Guangzhou, China; ^4^Deqing County Dexin Agricultural Development Co., Ltd., Zhaoqing, China

**Keywords:** methicillin-resistant *Staphylococcus epidermidis*, 2-methoxycinnamaldehyde, cinnamon, metabolomics, ROS, TCA cycle

## Abstract

Methicillin-resistant *Staphylococcus epidermidis* (MRSE) is one of the most commonly found pathogens that may cause uncontrollable infections in immunocompromised and hospitalized patients. Compounds isolated from cinnamon such as cinnamaldehyde and cinnamic acid showed promising anti-oxidant, anti-tumor, and immunoregulatory effects; more importantly, these compounds also possess promising broad-spectrum antibacterial activity. In this study, the potential antibacterial activity of 2-methoxycinnamaldehyde (MCA), another compound in cinnamon, against MRSE was investigated. Combining the broth microdilution test, live/dead assay, and biofilm formation assay, we found MCA was able to inhibit the proliferation, as well as the biofilm formation of MRSE, indicating MCA could not only affect the growth of MRSE but also inhibit the pathogenic potential of this bacterium. Additionally, the results of scanning electron microscopy (SEM) and transmission electron microscopy (TEM) demonstrated that MCA caused morphological changes and the leakage of DNA, RNA, and cellular contents of MRSE. Due to the close relationship between cell wall synthesis, ROS formation, and cell metabolism, the ROS level and metabolic profile of MRSE were explored. Our study showed MCA significantly increased the ROS production in MRSE, and the following metabolomics analysis showed that the increased ROS production may partially be due to the increased metabolic flux through the TCA cycle. In addition, we noticed the metabolic flux through the pentose phosphate pathway (PPP) was upregulated accompanied by elevated ROS production. Therefore, the alterations in cell metabolism and increased ROS production could lead to the damage of the cell wall, which in turn decreased the proliferation of MRSE. In conclusion, MCA seemed to be a promising alternative antimicrobial agent to control MRSE infections.

## Introduction

*Staphylococcus epidermidis* belongs to coagulase-negative staphylococci (Kloos and Musselwhite, 1975; Franca et al., 2021), which comprise a large group of related species commonly found on the surface of healthy persons. Like other opportunistic pathogens, *S. epidermidis* has long been regarded as an innocuous commensal colonizer on the human skin and functions as a bacterial barrier in protecting humans from the harmful pathogen (Cogen et al., 2010). Moreover, *S. epidermidis* is also an important immunoregulatory bacterium participating in tissue repair (Pastar et al., 2020). However, *S. epidermidis* is also a commonly found pathogen of catheter-related and indwelling medical devices (Otto, 2009; Wi et al., 2018). Among immunocompromised and hospitalized patients, *S. epidermidis* infection may lead to bacteremia and sepsis, especially in neonates (Otto, 2017). Although most of *S. epidermidis* is sensitive to commonly used antibiotics, the abuse of antibiotics in the food industry, inappropriate prescribing during clinical usage, the shrinking pool of novel available antibiotics (Ventola, 2015; Rani et al., 2021), and the emergence of antibiotic-resistant *S. epidermidis*, uncontrolled MRSE infection is becoming an urgent health problem.

Natural products and their derivatives have been considered promising sources for novel antibiotics development (Butler et al., 2014). Accumulating evidence demonstrates that essential oil and its components such as terpenes, alcohols, aldehydes, and ketones (Sharifi-Rad et al., 2017) can kill bacteria by affecting the zeta potential, increasing the permeability of the membrane, or inhibiting the flux system (Yang et al., 2021). Among the reported medicinal plants, cinnamon shows promising inhibitory effects against commonly known pathogens, such as bacteria, fungi, parasites, and viruses. For example, the essential oil of *Cinnamomum zeylanicum* shows broad-spectrum antibacterial effects against *Staphylococcus aureus*, methicillin-resistant *Staphylococcus aureus* (MRSA), *Streptococcus mutans*, *Lactobacillus casei*, *Candida tropicalis*, and *Candida glabrata* by inhibiting their proliferation and survival, as well as affecting the formation of biofilm (Budri et al., 2015; Yanakiev, 2020). Additionally, the essential oil from the bark or leaf of *C. zeylanicum* can decrease the hyphae formation, increase the cell permeability, and inhibit the growth or survival of *Candida albicans* and *Candida auris* (Tran et al., 2020).

MCA is another main bioactive ingredient isolated from *Cinnamomum zeylanicum*, *Cinnamomum cassia*, and *Cinnamomi ramulus* (Wu et al., 2014; Gunawardena et al., 2015; Li et al., 2021), which shows potent anti-oxidant, anti-inflammatory (Reddy et al., 2004; Hwa et al., 2012), anti-atherosclerotic (Jin and Kim, 2017), anti-tumor (Yamakawa et al., 2011), and antibacterial activities (Morozumi, 1978). Although the studies on the antibacterial effects of MCA against MRSE and the underline mechanism are quite limited, the analogs of MCA, such as cinnamaldehyde and coniferyl aldehyde, are demonstrated to possess promising metabolic regulatory and redox regulatory activity. Considering the structural similarity between MCA and its analogs, as well as the close relationship between ROS production, cell metabolism, and bacterial survival, the effects of MCA on the proliferation, the ROS level, and the metabolic profile of MRSE were investigated. Our study could pave the road for the discovery of novel antibiotics against MRSE, an extremely common pathogen in patients with chronic diseases (Brickner and Mobashery, 2007).

## Materials and Methods

### Strains and Cultures

Methicillin-resistant *Staphylococcus epidermidis* (*S. epidermidis* RP62A/ATCC 35984) was obtained from the American Type Culture Collection (ATCC, Manassas, VA, United States). MRSE was activated in the cation-adjusted MH broth (CAMHB, Qingdao Hope Biotechnology, Qingdao, China) at 37^°^C under aerobic conditions overnight. Bacterial strains were kept in the CAMHB with 20% glycerol at -80^°^C. Bacterial growth was measured by monitoring the OD_600_ using the UV–Vis spectrophotometer (Beijing Purkinje General Instrument Co., Ltd., Beijing, China). The bacteria in the logarithmic phase (OD_600_ 0.6–1.2) were collected and used throughout the following experiments.

### Determination of the Minimum Inhibitory Concentration and Minimal Bactericidal Concentration

2-Methoxycinnamaldehyde was purchased from Macklin (Macklin, Shanghai, China), and vancomycin was purchased from BioFoxx (BioFoxx, Einhausen, Germany). To minimize the influence of the solvent on the growth and proliferation of MRSE, a MAC stock solution was prepared with ethyl alcohol (Damao, Tianjin, China), and 0.1% ethyl alcohol was used as vehicle control throughout the following experiments. The minimum inhibitory concentration (MIC) and minimal bactericidal concentration (MBC) of MCA against MRSE were determined using the broth microdilution test according to the guidelines of the Clinical and Laboratory Standards Association 2017 (CLSI, 2017). Briefly, 50 μL of the bacterial suspension diluted to 2 × 10^5^CFU/mL was inoculated in a 96-well plate and treated with different concentrations of MCA (Macklin, Shanghai, China), ranging from 55 to 1,760 μg/mL, while 0.1% ethyl alcohol was used as the vehicle control, and vancomycin was used as the positive control (concentrations ranging from 0.5 to 8 μg/mL). After incubation at 37^°^C for 24 h, the MIC was determined when the lowest concentration (in μg/mL) of MCA showed inhibitory effects on the growth of MRSE. Subsequently, 10 μL cell suspension from each well was seeded in the Mueller–Hinton agar plates at 37^°^C for 24 h, and the MBC was determined when the lowest concentration of MCA inhibits the formation of the visible colony in the plates.

### Growth Assay

The growth curve was used to assess the antibacterial effect of the MCA. MRSE in the logarithmic phase diluted to ∼10^6^ CFU/mL was treated with MCA at 1/2× MIC and 1× MIC, while 0.1% ethyl alcohol was used as a vehicle control. Then, the growth of MRSE was evaluated by monitoring the OD_600_ values of bacteria incubated for 0, 2, 4, 6, 8, 10, 12, and 24 h at 37^°^C and shaken at 150 rpm.

### Biofilm Formation Assay

The biofilm formation assay of MRSE was performed based on previous reports (Coffey and Anderson, 2014; Piras et al., 2019) with some modifications. In brief, MRSE in the logarithmic phase (∼2 × 10^7^ CFU/mL, 100 μL/well) was seeded in a 96-well plate containing 100 μL/well of MCA at 1× MIC and 2× MIC, and the wells with 0.1% ethyl alcohol were used as the control. The cultures were incubated at 37^°^C for 24 h. After gently washing, the biofilms were stained with 1% crystal violet (CV, Beijing Leagene Biotech Co., Ltd., China) of 200 μL for 15 min. Subsequently, the excess CV was discarded, and the wells were washed three times with distilled water. After the plate was air-dried at RT, 200 μL of ethanol was added to each well for 20 min. Finally, OD_570_ was measured by using a microplate reader to quantify the biofilms.

### Determination of DNA and RNA in the Supernatant

The bacteria in the logarithmic growth phase were collected and resuspended in PBS with a desired density (OD_600_ = 1). Then, MRSE was treated with MCA at 1× MIC and 2× MIC for 6 h. Bacteria treated with 0.1% ethyl alcohol were used as the vehicle control. Subsequently, the supernatant filtered through a 0.22-μm Millipore filter was analyzed in the NanoDrop 2000 (ThermoFisher Scientific, Waltham, MA, United States) for determining the release of DNA and RNA (260 nm), as described previously (Spadari et al., 2018) with some modifications.

### Live/Dead Staining Assay

The viability of bacteria was assessed by using a Live/Dead BacLight Bacterial Viability Kit (Invitrogen, Carlsbad, CA, United States) and an FV3000 confocal laser scanning microscope (CLSM, Olympus, Japan) as described in the literature (Wang et al., 2020). In brief, the bacteria in the logarithmic phase diluted to ∼1 × 10^8^ CFU/mL were treated with 0.1% ethyl alcohol (vehicle control) and 1× MIC and 2× MIC of MCA for 6 h. Then, the bacterial pellet was collected and washed with PBS three times. Afterward, the bacteria were incubated with a mixture containing SYTO™9 (5 μM, staining live cells) and propidium iodide (30 μM, staining dead cells) at room temperature for 20 min. Then, the stained bacteria were washed three times and imaged using CLSM with excitation wavelengths at 488 and 560 nm. The 70% EtOH was employed as the positive control.

### Scanning Electron Microscopy Analysis

The morphology of MRSE treated with different concentrations of MCA was observed by SEM as previously reported (Palka et al., 2020). Briefly, the bacterial cells in the logarithmic phase were resuspended in the fresh medium at ∼10^8^ CFU/mL. Then, MRSE was incubated with MCA at 1× MIC and 2× MIC at 37^°^C for 6 h. After incubation, MRSE was washed three times with PBS and fixed overnight with 2.5% glutaraldehyde at 4^°^C. Subsequently, the samples were dehydrated with gradually increased concentrations of ethanol (50, 70, 90, 100, and 100%) for 15 min, respectively. The dehydrated samples were dried by using the critical point dryer for 2 h. Finally, the specimens were sputtered with gold, and morphological changes of MRSE were observed under SEM (SU8020, Hitachi, Japan).

### Transmission Electron Microscopy Analysis

The samples used for TEM analysis were prepared following the same procedure as SEM. In short, the treated bacterial cells were immobilized in 2.5% glutaraldehyde overnight, fixed with 1% osmic acid, washed with PBS, and dehydrated with the series concentration of ethanol. Then, the dehydrated samples were treated with embedding agents at 70^°^C overnight. The embedded samples were sliced into ultra-thin sections (70–90 nm), stained with the mixture of uranyl acetate and lead citrate, and finally observed by TEM (H-7650, Hitachi, Japan).

### Metabonomics

#### Metabolite Preparation and GC-MS Analysis

Metabolite preparation and derivatization were performed according to the literature (Chen et al., 2020; Kuang et al., 2021). Briefly, MRSE in the logarithmic phase was resuspended in the fresh medium at ∼2 × 10^8^ CFU/mL. After treating cells with 1× MIC for 6 h, the bacterial cells were quenched with liquid nitrogen before adding 1 mL prechilled methanol. The metabolites were extracted by using an ice bath ultrasound combined with repeated freeze–thaw. The supernatant containing the analytical internal standard was concentrated in a vacuum drying oven. The completely dried samples were resuspended in 80 μL of 20 mg/mL methoxyamine hydrochloride solution in pyridine at 37^°^C for 120 min, and then 80 μL of N-methyl-N-(trimethylsilyl) trifluoroacetamide (MSTFA) was added and incubated at 37^°^C for another 45 min. After centrifugation for 10 min at 12,000 rpm, the supernatant (about 1.0 μL) was subsequently loaded onto the Agilent HP-5ms GC columns by a splitless model, and the analysis was performed on the GC-MS (6890N/5973, Agilent, Santa Clara, CA, United States).

#### Data Analysis

After peak extraction, retention time correction, peak area integration, and deconvolution, the qualitative analysis and peak identification of metabolomic profile were based on matching with the NIST 17 library and the Fiehn library. The mass spectral data were normalized by internal standard and standardized by using the quartile method for further metabolomics analysis. Then, the differential metabolites were selected on the basis of the combination of a fold change compared to the control group, *p*-values from a two-tailed Student’s *t*-test on the normalized peak areas, and the adjusting *p*-value after correction. The PCA and OPLS-DA were carried out to visualize the metabolic alterations; in addition, the z-score plot was used to visualize the dispersion of identified differential metabolites. The fold change of differential metabolites in the control and MCA treatment groups was visualized by heatmap (Supplementary Figure 1). The patterns of correlations between metabolites were analyzed by Pearson’s correlation, and correlation coefficients were plotted as heatmaps. The PCA and OPLS-DA were performed by SIMCA 14.1, and the heatmaps were completed by R software. Finally, the differential metabolites were mapped to the KEGG pathway database, and metabolic pathways were enriched by the enrichment analysis module in MetaboAnalyst 5.0.^1^

### Determination of Enzyme Activities and Adenosine 5’-Triphosphate

The bacteria incubated with MCA at 1× MIC were harvested and broken down by cellular ultrasound on an ice bath. Subsequently, malic dehydrogenase (MDH), citrate synthase (CS), succinic dehydrogenase (SDH), α-ketoglutarate dehydrogenase (α-KGDH), isocitrate dehydrogenase (ICDH), glucose-6-phosphate dehydrogenase (G6PDH) activities, and adenosine 5′-triphosphate (ATP) content were measured by using the corresponding kits as per the instructions, respectively. The assay kits for all key enzymes were purchased from Shanghai Enzyme-linked Biotechnology Co., Ltd. (Shanghai, China). The ATP assay kit was purchased from Beyotime Biotechnology (Shanghai, China).

### Determination of ROS by Flow Cytometry Analysis

The ROS level in bacteria after MCA treatment was measured by CM-H_2_DCFDA staining as described in the literature (Dong et al., 2015). After treating bacteria with MCA of the indicated concentration, the cell pellet was collected and stained with 2 μM CM-H_2_DCFDA for 45 min at 37^°^C. After washing with HBSS, the cell suspension was analyzed by flow cytometry, and the median fluorescence intensity (MFI) was used for quantification.

### Statistical Analysis

All data were obtained from at least three independent experiments and represented as mean ± SEM, and the pooled data were analyzed by using Student’s *t*-test by SPSS 19.0 software, except the data from metabolomics. *p* < 0.05 was considered statistically significant.

## Results

### MCA Inhibited the Growth and Biofilm Formation and Increased the Permeability of Methicillin-Resistant *Staphylococcus epidermidis*

**FIGURE 1:**
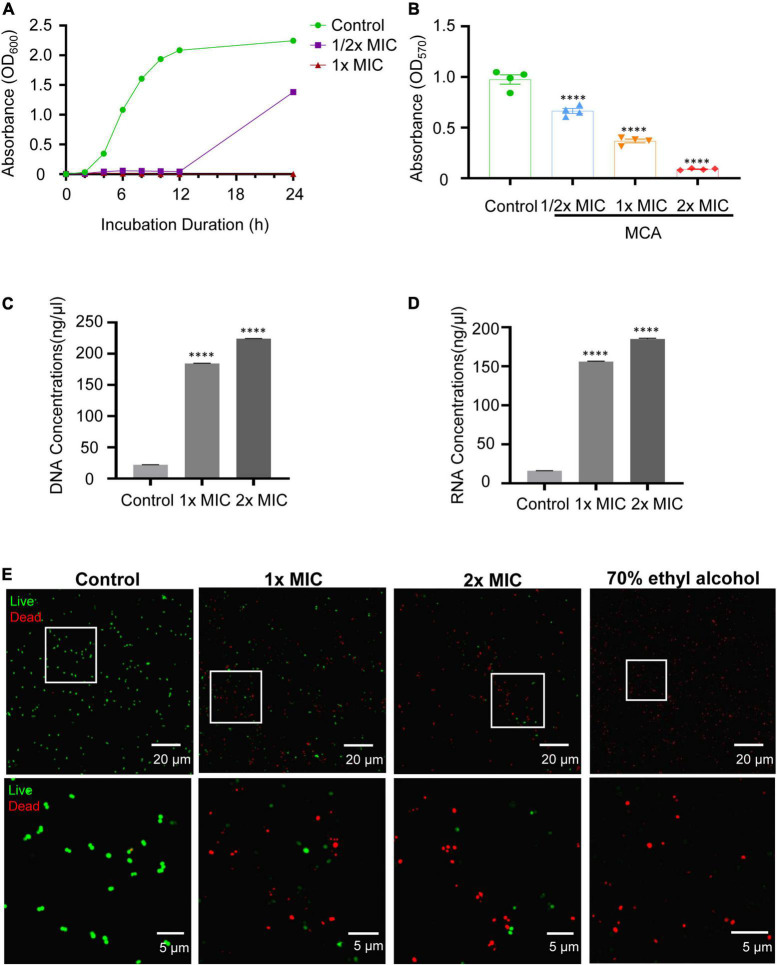
MCA inhibited the growth and biofilm formation, and increased the permeability of MRSE. **(A)** MCA inhibited the proliferation of MRSE in a dose-dependent way. MRSE in the exponential growth phase was treated with MCA at 0, 1/2× MIC, and 1× MIC for the indicated time, respectively, while 0.1% EtOH was used as the vehicle control. The proliferation of MRSE was measured by monitoring the absorbance at 600 nm (OD600). **(B)** MRSE inhibited biofilm formation of MRSE. MRSE in the exponential growth phase was treated with MCA at the indicated concentration for 24 h, and the biofilm formation was measured by monitoring the absorbance at 570 nm (OD570) after CV staining. **(C,D)** MCA induced the leakage of DNA and RNA from MRSE. MRSE in the exponential growth phase was treated with MCA at 1× MIC and 2× MIC for 6 h, while the 0.1% EtOH was used as vehicle control. The leakages of DNA and RNA were quantified by measuring the absorbance at 260 and 230 nm using a NanoDrop spectrophotometer. The pooled data are presented as mean ± SEM of at least three independent experiments. *p < 0.05, ^**^p < 0.01, ^***^p < 0.001, ^****^p < 0.0001. **(E)** MCA decreased the viability of MRSE. MRSE was treated with MCA at the indicated concentration for 6 h and then stained with SYTO-9/PI. The images were obtained by confocal microscopy, and the green and red fluorescence indicate the live and dead bacteria, respectively. The highlighted regions are magnified in the lower panel. The representative images were selected from at least three independent experiments.

To evaluate the potential effect of MCA on the proliferation of MRSE, MIC, and MBC, and growth assays were employed. The results showed that the MIC and MBC of MCA on MRSE were 220 and 880 μg/mL, respectively. Vancomycin was used as the positive control, and the MIC and MBC of vancomycin on the bacteria were 1 μg/mL. As shown in Figure 1A, the absorbance at 600 nm began to increase after incubation for 2 h in the control group, indicating the vehicle control did not affect the proliferation of MRSE (Kerk et al., 2018). In the presence of MCA at 1/2× MIC, the absorbance at 600 nm began to increase until 12 h. However, the absorbance at 600 nm did not increase during the whole experiment. The results indicated that MCA inhibited the proliferation of MRSE in a dose-dependent manner.

The biofilm formation, another key factor contributing to the pathogenesis of MRSE, was investigated. The ability of biofilm formation was quantified by the CV staining assay. As shown in Figure 1B, MCA treatment decreased the absorbance at OD_570_, which was commonly used as an indicator for biofilm formation by MRSE, in a dosage-dependent way. In particular, biofilm formation was reduced by more than 60% at 1× MIC. In the presence of MCA at 2× MIC, the absorbance at OD_570_ decreased to nearly baseline level (OD_570_ was < 0.1), which is comparable to the blank control (Ranjith et al., 2017). The significantly decreased absorbance indicated that the biofilms were not formed at 2× MIC for 24 h.

In addition, we found the permeability of MRSE was increased after MCA treatment. As shown in Figure 1C, after incubating MRSE cells with the MCA at 37^°^C for 6 h, the DNA content of MRSE in the supernatant was significantly higher than that of the control group in a dose-dependent manner. The result of RNA leakage was in agreement with the DNA leakage data (Figure 1D). Additionally, the live/dead assay was employed to monitor the viability of bacterial populations as a function of the membrane integrity. As shown in Figure 1E, MRSE emitted strong green fluorescence after treating with the vehicle control (0.1% EtOH), indicating almost all of the bacteria were alive and possessed an intact membrane. However, the bright green fluorescence was gradually replaced with red fluorescence when treated with MCA. In the presence of MCA at 1× MIC, around half of the cells were stained with red fluorescence, indicating the emergence of damaged cell walls, as well as the dead cells. As the MCA concentration increased, the majority of the cells emitted bright red fluorescence when treated with MCA at 2× MIC, indicating the accumulation of damaged bacteria; 70% ethyl alcohol was used as positive control, and all cells emitted bright red fluorescence, indicating nearly all bacteria were damaged or died.

### MCA Altered the Morphology and Ultrastructure of Methicillin-Resistant *Staphylococcus epidermidis*

**FIGURE 2:**
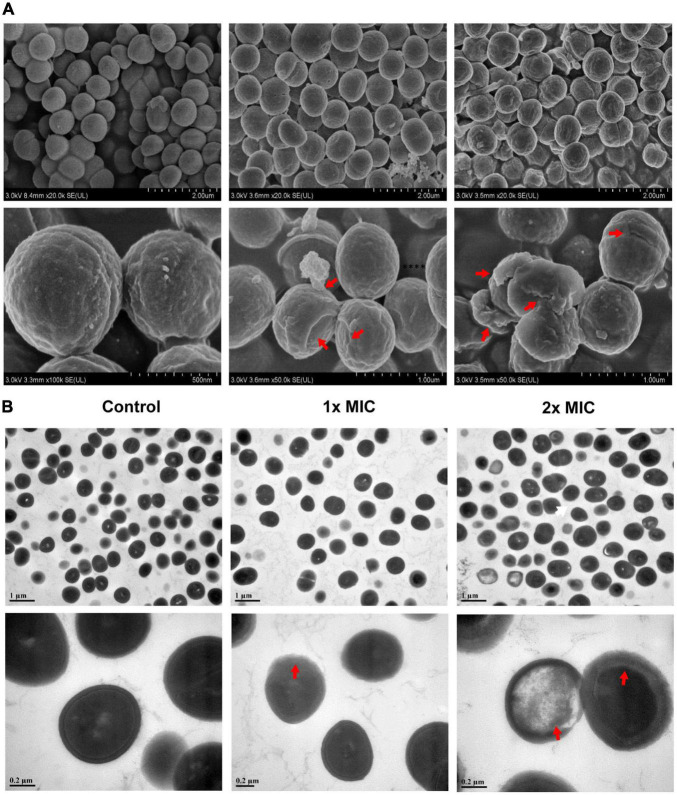
MCA altered the morphology and ultrastructure of MRSE. After treatment with MCA of indicated concentrations for 6 h, the bacteria were collected, fixed, and prepared for electron microscopy analysis. The images from SEM and TEM are shown in panels **(A,B)**, respectively. The scale bar in panel **(B)** indicate 1 and 0.2 μm in the upper and lower panels, respectively. The indications of cell membrane damage, corrugation, shriveling, and the leakage of the cytoplasm are highlighted with red arrows.

Considering that cell wall integrity played an essential role in bacterial growth and biofilm formation, the morphological and ultrastructural alterations of MRSE after MCA treatment were analyzed *via* SEM and TEM (Figure 2). As shown in Figure 2A, the cell membranes and walls of MRSE in the control group were smooth and intact. However, the MCA treatment caused severe morphological damages to MRSE. For example, after treatment with MCA at 1× MIC, the surface of a small number of bacteria was slightly corrugated and shriveled. In addition, we observed the leakage of their intracellular contents. As expected, the majority of bacterial cells displayed severe surface collapse and morphological destruction after treatment with MCA at 2× MIC, or even broken into debris. The results from TEM analysis revealed that the cytoplasm of untreated MRSE was homogeneously distributed and well protected by the complete bacterial membranes (Figure 2B). By contrast, the MCA-treated bacteria clearly showed cell membrane damage and the leakage of cytoplasm, which was characterized by reduced intracellular contents and conspicuous cytoplasmic zones, in a dose-dependent manner.

### Multivariate Statistical Analysis of Metabolic Fingerprinting Identified 11 Biomarkers

**FIGURE 3:**
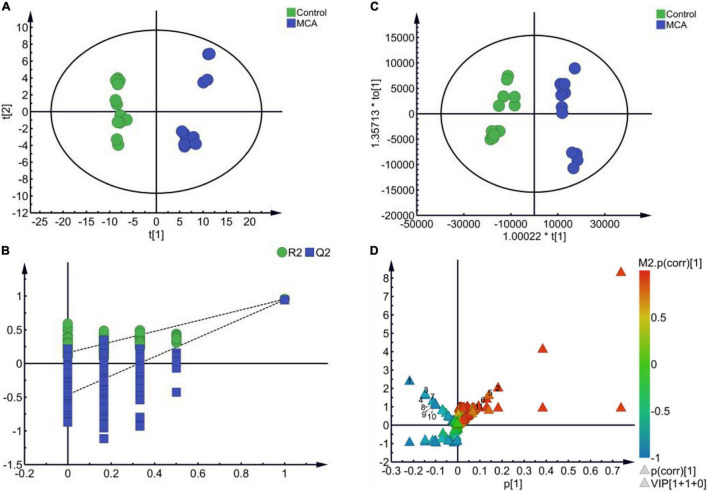
Multivariate statistical analysis. **(A)** PCA score plots (R2X = 0.675, Q2 = 0.593). **(B)** OPLS-DA score plots (R2X = 0.909, R2Y = 0.954, Q2 = 0.944). **(C)** 200 permutation tests of PLS-DA. **(D)** (V + S) plot of OPLS-DA.

Cell metabolism not only functioned as a direct reflection of the enzymatic pathways and networks encoded within the genome but also provided a clearer picture for understanding alterations in the cell phenotype and physiology (Tang, 2011; Riedelsheimer et al., 2012). In our study, metabolomics was employed to investigate the effects of MCA on bacterial metabolism, as well as its potential mechanism. The metabolic profile of MRSE in the vehicle control and MCA group was constructed *via* GC-MS-based metabolomics from six biological replicates with two technical replicates. To identify the putative metabolic biomarkers, the total 124 identified metabolites were subjected to multivariate statistical analysis. As shown in Figure 3, PCA and OPLS-DA showed a clear separation between control and MCA groups (Figures 3A,B). The results of PCA (R2X = 0.675, Q2 = 0.593) and OPLS-DA (R2X = 0.909, R2Y = 0.954, Q2 = 0.944) proved the reliable predictability of the models. The OPLS-DA model was further tested the robustness and overfitting by performing the permutation test (n = 200 times) (Figure 3C). Furthermore, metabolites of MRSE were visualized in the (V + S) scatter plot (Figure 3D). In general, the VIP values reflected the influence of each variable, while the longer the distance, the greater the impact value. Combining variable importance in projection (VIP score > 1.00), the absolute value of correlation [*p* (corr) > 0.50], and the *t*-test (*p* < 0.050), 11 putative biomarkers with the most contribution were identified (Table 1). The identified biomarkers included L-glutamate, D-alanyl-D-alanine, L-proline, sn-glycerol 3-phosphate, 5-aminopentanoate, L-lysine, L-threonine, L-alanine, 5,6-dihydrouracil, L-serine, and lactate, while four of them increased and seven of them decreased compared to the control group.

**TABLE 1:** Identified metabolic biomarkers.

Number	Name	OPLS-DA (VIP)	OPLS-DA [p(corr)]	remark
1	L-Glutamate	2.36931	0.946563	****
2	D-Alany1-D-alanine	2.02014	0.921418	****
3	L-Proline	1.67139	0.829538	****
4	sn-Glycerol-3-phosphate	1.60897	0.929770	****
5	5-Aminopentanoate	1.59915	0.795938	****
6	L-Lysine	1.40204	0.945469	****
7	L-Threonine	1.28046	0.844373	****
8	L-Alanine	1.12990	0.857487	****
9	5,6-Dihydrouracil	1.09775	0.924627	****
10	L-Serine	1.06673	0.966028	****
11	Lactate	1.01673	0.941555	****

The thresholds of VIP score, p (corr) calculated from OPLS-DA, and p-value calculated from t-test were set to 1.00, 0.50, and 0.05, respectively. *p < 0.05, **p < 0.01, ***p < 0.001, ****p < 0.0001.

### MCA Induced Remarkable Alteration of the Metabolic Profile Within Methicillin-Resistant *Staphylococcus epidermidis*

**FIGURE 4:**
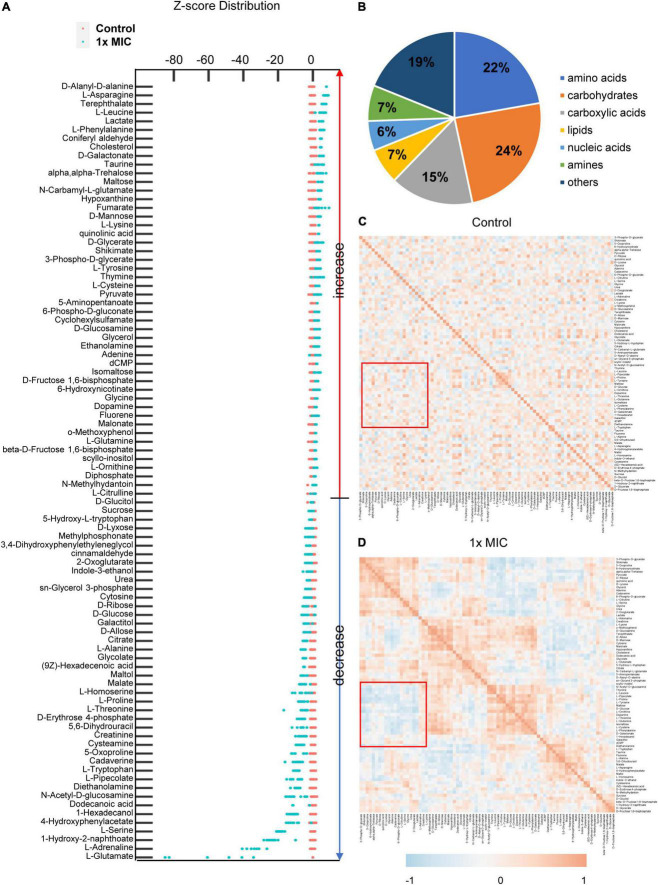
MCA induced remarkable alterations of metabolic profile in MRSE. **(A)** z-score distribution of the control and the MIC group. The peak areas of identified differential metabolites were normalized by the ribitol, and the z-score of each metabolite was calculated based on the mean and standard deviation of the control group. The classification of differential metabolites is shown in panel **(B)**, and the correlations of differential metabolites in the control and MIC groups are plotted in panel **(C)**. The difference of correlation between saccharide and amino acid metabolism is highlighted with the red square. The correlations of differential metabolites in the control and MIC groups were calculated from six independent experiments by Pearson’s correlation function. The heatmap of correlation and z-score plot were calculated and plotted with R statistical computing environment.

After screening metabolites by the fold change of the peak area of each identified metabolites and the *p*-value, 90 differential metabolites were selected, and Z-score plots of these metabolites in two groups relative to their corresponding control groups are shown in Figure 4A. The identified differential metabolites belonged to seven classes, including amino acids (22%), carbohydrates (24%), carboxylic acids (15%), lipids (7%), nucleic acids (6%), amines (7%), and others (17%) based on the biological roles (Figure 4B). To better understand the relationships between differential metabolites in the control and MCA group, Pearson’s correlation method was employed (Steuer et al., 2003; Kotze et al., 2013). The correlation coefficients for metabolites were plotted as the heatmap. In the heatmap, the negative correlations were colored blue, while the positive correlations were colored red. Considering the identified biomarkers mainly categorized as amino acids and carbohydrate, the regions describing the correlation between glucose and amino acid metabolism was highlighted. As shown in Figures 4C, A positive correlation between glucose and amino acid metabolism was observed in the control group. By contrast, negative correlations between them were observed in the 1× MIC group (Figure 4D), indicating that the amino acid metabolism was no longer synchronized with glucose metabolism in the presence of MCA.

### MCA Mainly Affected the TCA Cycle, Amino Acid Metabolism, and Pentose Phosphate Pathway in Methicillin-Resistant *Staphylococcus epidermidis*

**FIGURE 5:**
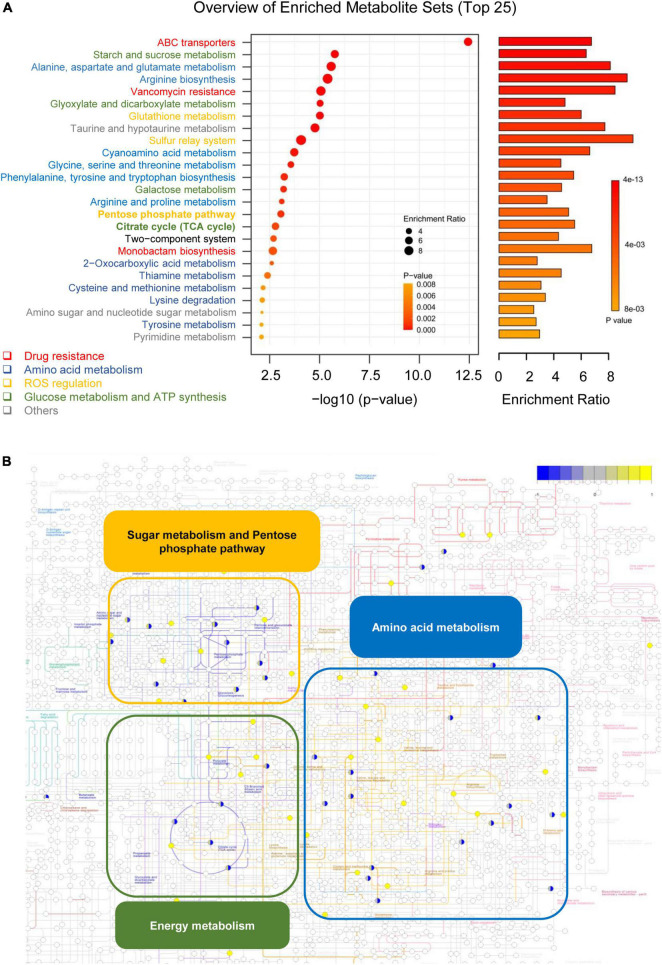
MCA mainly affected the metabolic flux of the TCA cycle, amino acid metabolism, and pentose phosphate pathway in MRSE. **(A)** Putative signaling pathways affected by MCA were analyzed by enrichment analysis. The enrichment analysis was performed by MetaboAnalyst 2.0 software (http://www.metaboanalyst.ca/) with identified differential metabolites and a reference dataset extracted from KEGG (organism: Ser), and the top 25 pathways were extracted for visualization. The pathways belonged to drug resistance, amino acid metabolism, ROS regulation, glucose metabolism, ATP synthesis, and others are colored red, dark blue, orange, grass green, and gray, respectively. **(B)** Overview of differential metabolites involved in pathways. The differential metabolites were mapped to the overview of the metabolic pathways (ser: 01100 in KEGG database). The up- and downregulated metabolites are colored yellow and blue, respectively.

To further understand the biological functions of key metabolites, KEGG pathway analysis was used. As shown in Figure 5A, the metabolite pathways responsible for drug resistance (such as ABC transporters, vancomycin resistance, and monobactam biosynthesis), amino acid metabolism (such as alanine, aspartate, and glutamate metabolism), ROS generation, and glucose metabolism were identified. As shown in Figure 5B, the differential metabolites identified in metabolomics were mapped to the overview of metabolic pathways (ser: 01100 in KEGG database). The upregulated metabolites and downregulated metabolites were represented as the yellow and blue circles, respectively.

### MCA Treatment Increased Metabolic Flux Through the TCA Cycle and the Intracellular ROS

**FIGURE 6:**
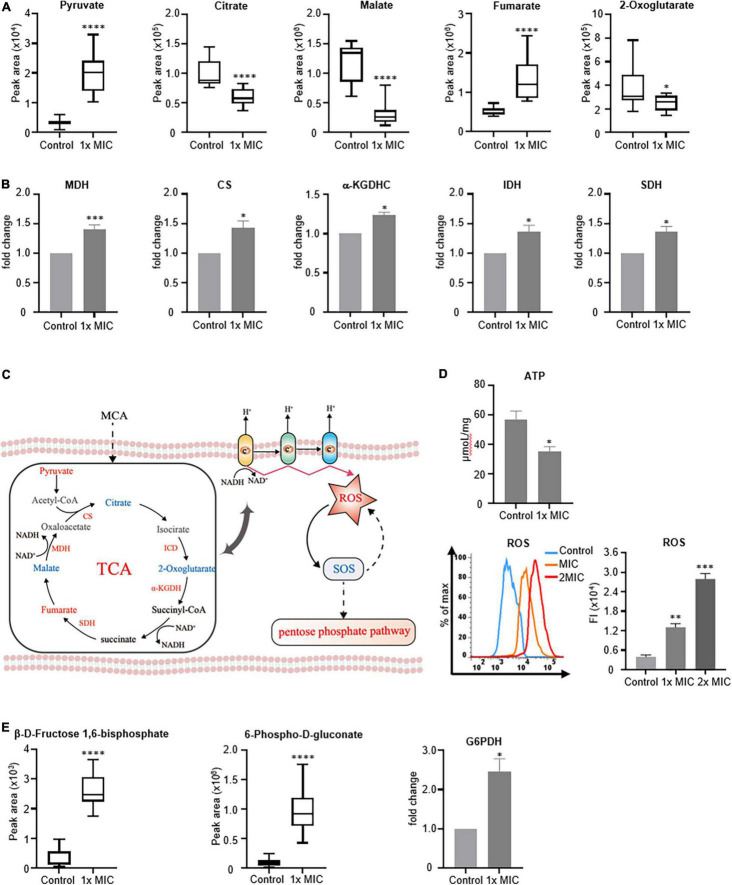
MCA treatment increased metabolic flux through the TCA cycle and the intracellular ROS. MRSE in the logarithmic growth phase was treated with MCA at 1× MIC for 6 h, and 0.1% ethanol was used as the vehicle control. **(A)** Peak areas of key metabolites in the TCA cycle of control and MCA group. The peak areas of indicated metabolites were quantified by GC-MS-based metabolomics. **(B)** Activities of key enzymes catalyzed the irreversible steps of the TCA cycle. After treatment, cell pellets were lysed, and the activities of enzymes were analyzed by ELISA kits. After normalizing the measured activities to the control group, the fold changes were used for quantification. **(C)** Illustration of connections between TCA and ROS generation. **(D)** ATP content and ROS level of MRSE. After treatment, the intracellular ATP was quantified by using a chemiluminescence kit, and the intracellular ROS was analyzed by CM-H_2_DCFDA staining. The histogram of fluorescence from flow cytometry was plotted, and the medium fluorescence intensity was used for quantification. **(E)** Activities of key enzymes involved in the pentose phosphorylation pathway. After treatment, the activities of key enzymes involved in PPP were assayed using the ELISA kit. After normalizing the measured activities to the control group, the fold changes were used for quantification. The pooled data are presented as mean ± SEM of at least three independent experiments. *p < 0.05, ^**^p < 0.01, ^***^p < 0.001, ^****^p < 0.0001.

The TCA cycle is a primary metabolic pathway utilized by aerobic organisms to generate not only the energy required by the cells but also the intermediates for biosynthetic pathways. To better understand the effects of MCA on the metabolism of MRSE, the accumulation of metabolites, as well as the activities of key enzymes that participated in the TCA cycle, was analyzed. As shown in Figure 6A, MCA treatment decreased the accumulation of intermediate, such as malate, 2-oxoglutarate, and citrate but increased the intracellular concentration of fumarate, indicating the metabolic flux through the TCA cycle was affected. To further investigate the alteration of MCA on the TCA cycle in bacterial metabolism, the activities of MDH, CS, SDH, α-KGDHC, and IDH were investigated. As shown in Figure 6B, the activities of CS, α-KGDHC, and IDH increased by approximately 50%. Considering these enzymes catalyzing the irreversible steps in the TCA cycle, the results of our study indicated the metabolic flux through the TCA cycle was increased.

Because the TCA cycle participated in not only energy metabolism but also ROS production (Figure 6C), the intracellular ATP content, as well as the ROS level, was investigated. As shown in Figure 6D, compared to the control group, the content of ATP decreased by approximately 37.60%, but the intracellular ROS increased by approximately eightfold in the presence of MCA at 2× MIC. Additionally, we found that the concentration of intermediates involved in the pentose-phosphate pathway increased, indicating metabolic flux through PPP was upregulated. Accordingly, G6PDH, the key enzyme in the pentose phosphate pathway, was investigated. As shown in Figure 6E, the activity of G6PDH in the MCA-treated group increased by approximately 150%, implying that PPP was activated by MCA, which was consistent with previous pathway results.

## Discussion

In our study, we found MCA possessed promising antibacterial activity against MRSE. The cell wall of bacteria is vital to their survival and pathogenic potential. Thus, targeting the cell wall of bacteria is still one of the most commonly used strategies for antibiotics development (Jovetic et al., 2010). By employing live/death staining, SEM, and TEM, we found MCA could affect the integrity and permeability of the cell wall of MRSE, lead to the leakage of DNA and RNA, and finally inhibit cell proliferation. Moreover, the maintenance of cell shape, structural integrity, and the cell wall also plays a critical role in regulating their biological functions and pathogenic potentials, such as attachment and biofilm formation (Huang et al., 2008; Dorr et al., 2019). As expected, MCA inhibited the biofilm formation of MRSE, indicating MCA not only inhibited the proliferation of MRSE but also held the potential for the purposeful use of decreasing the pathogenesis of MRSE.

Comparing the chemical structure between MCA and already identified antibiotics that targeted bacterial cell wall synthesis (Bush, 2012; Sarkar et al., 2017), we found the key structural motif, such as phosphoenolpyruvate (Hrast et al., 2014), β-lactam, or glycopeptides, was missing in MCA. As a result, it was very slightly possible that MCA directly targets the enzyme involved in cell wall synthesis. Although there are very few studies on MCA and its related targets, accumulating evidence shows the analogs of MCA, like cinnamaldehyde and cinnamic acid, possess promising metabolism regulatory activities, which may finally affect the synthesis of the cell wall of MRSE. For example, cinnamaldehyde could decrease glucose–lipid metabolism in human and murine adipose tissue (Jiang et al., 2017). Similarly, Zhang et al. demonstrated cinnamaldehyde could promote the utilization of glycogenic amino acids and the biosynthesis of the intermediates in the tricarboxylic acid (TCA) cycle by function as a covalent inhibitor of α-enolase (Zhang et al., 2020). Moreover, some studies demonstrated cinnamaldehyde possesses promising antibacterial activity by targeting 1,3-β-D-glucans and decreasing the cell wall integrity (Liu et al., 2019). Considering the synthesis of cell walls involved in saccharide, lipid, and amino acid metabolism, we hypothesize that altering the cell metabolism might be the potential mechanism of MCA. In line with the aforementioned studies, we found MCA was able to decrease the ATP level within MRSE, further indicating the interruption of cell metabolism. To better understand the potentially detailed mechanism of MCA from the perspective of cell metabolism, metabolomics was employed. We found MCA was able to interrupt the metabolism of amino acid, especially hydrophobic amino acid such as leucine, isoleucine, and valine, in MRSE. Maintaining a relatively high hydrophobicity of cell walls not only affected the pathogenicity and biofilm formation in Gram-positive bacteria (Reifsteck et al., 1987; Krasowska and Sigler, 2014) but also contributed to the cell wall integrity of bacteria. As a result, MCA might affect the integrity of the cell wall, cause morphological alteration, and decrease the proliferation of MRSE partially by interrupting amino acid metabolism, especially the hydrophobic amino acid.

In addition to decreasing the integrity of the cell membrane and cell wall, the results of our study demonstrated MCA increased the ROS level in MRSE. ROS-induced stress could cause the damage of DNA, RNA, lipid, and protein and lead to cellular structure changes and then bacterial cell death (Behera et al., 2019). As an alternative strategy for novel antibacterial drug development, the redox-active natural products or their derivatives were considered not only broad-spectrum enhancers of existing antimicrobial agents but also promising candidates to overcome the drug resistance (Khodade et al., 2014; Song et al., 2020). Although the induction of early ROS production was believed to trigger the activation of conserved SOS-related signaling pathways in bacteria to repair DNA damage, which in turn promoted the survival of bacteria (Rowe et al., 2020), the persistent increased ROS level would become self-sustaining or self-amplifying events and finally lead to cell death (Hong et al., 2019). For example, the persistent increased level of hydroxyl radicals could break DNA, increase the accumulation of lipoperoxides and carbonylated proteins, and disrupt the DNA repairing process by oxidizing dGTP and dCTP pools (Dwyer et al., 2015). Accordingly, we found MCA treatment was able to increase ROS levels after 6 h. The persistent ROS accumulation might affect not only the permeability of MRSE by disrupting the structure of the cell membrane and cell wall but also the survival of MRSE.

Alterations in metabolic flux could lead to increased ROS production. In MRSA, redirecting metabolism to the tricarboxylic acid (TCA) cycle would finally lead to the accumulation of NADH as an intermediate and increased ROS production (Thomas et al., 2013; Rosato et al., 2014). In the current study, we found MCA treatment could alter the concentration of intermediates found in the TCA cycle. MCA treatment also led to the increased activity of key enzymes in the TCA cycle, indicating the increased metabolism through this pathway. Accompanying with the increased metabolism through the TCA cycle, NADH was accumulated. In bacteria, NADH generated by the TCA cycle could be used for generating and maintaining the proton-motive force, which was finally linked to ATP production. On the other hand, complex I in bacteria could catalyze NADH oxidation and generate ROS (Vinogradov and Grivennikova, 2016). In our study, we found the ATP levels of MRSE were decreased after MCA treatment, and the metabolome flux through the TCA cycle was negatively correlated with ATP production, suggesting that NADH generated through the TCA cycle might be used for ROS production.

Different from previous studies that commonly consider MCA as an anti-oxidant because of its conjugate system and aldehyde group, the results of our study demonstrated the role of MCA in inducing ROS production in MRSE. One of the possible explanations could be MCA was able to react with glutathione, and the depletion of GSH would lead to the increase in the ROS level. However, the effects of MCA on the ROS production and the usage of NADH, especially its upstream target, still need further investigation.

In summary, the results of the current study demonstrated MCA was able to decrease the survival and proliferation of MRSE partially through its metabolic regulatory and redox regulatory activities. MCA treatment interrupted the correlation of glucose–amino acid metabolism and mediated disruption of the amino acid level. On the other hand, MCA redirected the metabolism of MRSE toward the TCA cycle, which resulted in increased ROS production. The combinatory effects of interrupted metabolism and enhanced ROS generation finally led to the decreased cell membrane and cell wall integrity, the leakage of DNA and RNA, and the decreased proliferation of MRSE. The results of our study may shed new light on the antibacterial mechanism of MCA and the discovery of novel antibacterial agents.

## Data Availability Statement

The raw data supporting the conclusions of this article will be made available by the authors, without undue reservation.

## Author Contributions

CQ conceptualized the project and conducted the experiments. CQ and LJ performed the formal analysis and wrote the manuscript. LZ, YZ, JC, XX, PD, and RL contributed to discussing the results and critical review of the manuscript. DY reviewed and edited the manuscript, provided supervision and project administration, and acquired funding. ZZ conceptualized the project, reviewed, provided supervision and project administration, edited the manuscript, and acquired funding. All authors made significant contributions to this article and participated actively in the conception and design of the experiments, read, and approved the final manuscript.

## Conflict of Interest

RL was employed by Deqing County Dexin Agricultural Development Co., Ltd. The remaining authors declare that the research was conducted in the absence of any commercial or financial relationships that could be construed as a potential conflict of interest.

## Publisher’s Note

All claims expressed in this article are solely those of the authors and do not necessarily represent those of their affiliated organizations, or those of the publisher, the editors and the reviewers. Any product that may be evaluated in this article, or claim that may be made by its manufacturer, is not guaranteed or endorsed by the publisher.

## Footnotes

1
https://www.metaboanalyst.ca/
