# Supplementary material for: Metabolomics-Driven Exploration of the Antibacterial Activity and Mechanism of 2-Methoxycinnamaldehyde
Source: Front Microbiol. 2022 Jul 7;13:864246. doi: 10.3389/fmicb.2022.864246 (PMC9301309; doi:10.3389/fmicb.2022.864246)
Supplement: Supplementary file 1 [file Image_1.pdf]

frontiers

Supplementary Material

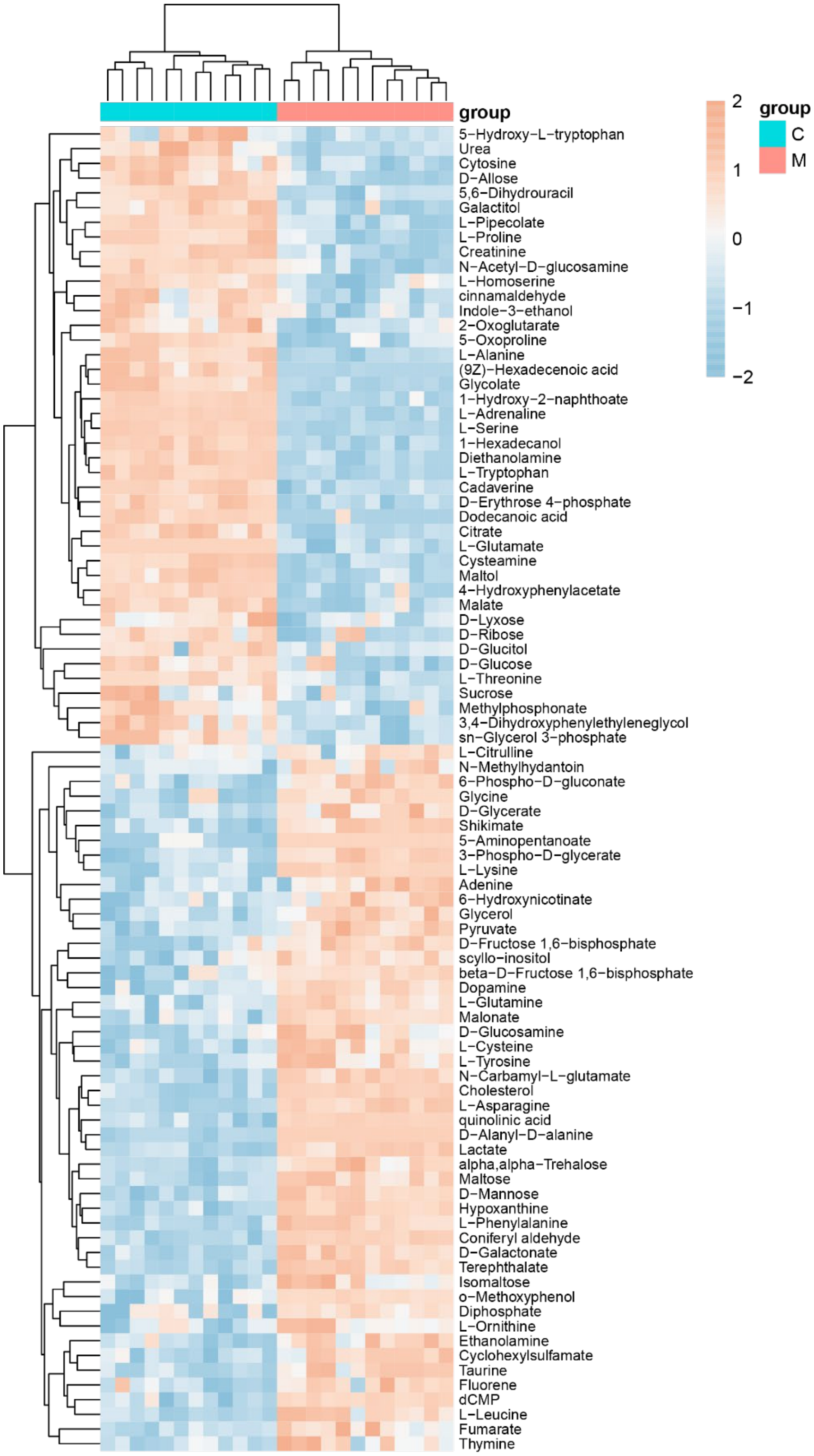

Supplementary Figure S1. The fold change of differential metabolites in the control and MCA treatment group. The control and MCA treated groups were colored cyan and red, respectively. In the

heatmap, the upregulated metabolites and down-regulated metabolites were colored light sky blue and light red, respectively.
